# Understanding spatiotemporal coupling of gene expression using single molecule RNA imaging technologies

**DOI:** 10.1080/21541264.2023.2199669

**Published:** 2023-04-12

**Authors:** Alan Gerber, Sander van Otterdijk, Frank J. Bruggeman, Evelina Tutucci

**Affiliations:** a Amsterdam UMC, Location Vrije Universiteit Amsterdam, Department of Neurosurgery, Amsterdam, The Netherlands; b Cancer Center Amsterdam, Brain Tumor Center Amsterdam, Amsterdam, The Netherlands; c Systems Biology Lab, A-LIFE department, Amsterdam Institute of Molecular and Life Sciences (AIMMS), Vrije Universiteit Amsterdam, Amsterdam, The Netherlands

**Keywords:** Gene expression coupling, single-molecule RNA imaging, transcription-translation coupling, eukaryotic gene expression, prokaryotic gene expression, RNA localization

## Abstract

Across all kingdoms of life, gene regulatory mechanisms underlie cellular adaptation to ever-changing environments. Regulation of gene expression adjusts protein synthesis and, in turn, cellular growth. Messenger RNAs are key molecules in the process of gene expression. Our ability to quantitatively measure mRNA expression in single cells has improved tremendously over the past decades. This revealed an unexpected coordination between the steps that control the life of an mRNA, from transcription to degradation. Here, we provide an overview of the state-of-the-art imaging approaches for measurement and quantitative understanding of gene expression, starting from the early visualizations of single genes by electron microscopy to current fluorescence-based approaches in single cells, including live-cell RNA-imaging approaches to FISH-based spatial transcriptomics across model organisms. We also highlight how these methods have shaped our current understanding of the spatiotemporal coupling between transcriptional and post-transcriptional events in prokaryotes. We conclude by discussing future challenges of this multidisciplinary field.

**Abbreviations:** mRNA: messenger RNA; rRNA: ribosomal rDNA; tRNA: transfer RNA; sRNA: small RNA; FISH: fluorescence *in situ* hybridization; RNP: ribonucleoprotein; smFISH: single RNA molecule FISH; smiFISH: single molecule inexpensive FISH; HCR-FISH: Hybridization Chain-Reaction-FISH; RCA: Rolling Circle Amplification; seqFISH: Sequential FISH; MERFISH: Multiplexed error robust FISH; UTR: Untranslated region; RBP: RNA binding protein; FP: fluorescent protein; eGFP: enhanced GFP, MCP: MS2 coat protein; PCP: PP7 coat protein; MB: Molecular beacons; sgRNA: single guide RNA.

## Introduction

Gene expression is a multistep process starting with transcription initiation and ending with protein degradation. So far, the various phases of gene expression have been mainly studied as separate events, but increasing evidence suggests that they are coupled, implying that the control of one stage influences the stage before and after it. For instance, in prokaryotic cells, translation can occur co-transcriptionally, while in eukaryotic cells, pre-mRNA splicing mainly occurs co-transcriptionally, and cytoplasmic mRNA localization was shown to influence translation efficiency and possibly decay [1–5].

Much of our knowledge about the regulation of RNA synthesis and processing comes from ensemble biochemical or genetic experiments which assess the average behavior of a large number of molecules across a population of cells. At the end of the 1960s – when biochemical studies were playing a central role in uncovering and characterizing the main actors and the molecular basis of the expression of genes – a technological breakthrough enabled the acquisition of images of single genes caught in the act of transcription [6]. The ability to take such snapshots of active genes facilitated the investigation of questions related to gene transcription and its coupling to processes associated with gene expression, which would have otherwise been difficult to address in cell populations. The true revolution started with the development of single-cell and single-molecule RNA imaging approaches at the end of the 1990s [7], which provided new quantitative tools to study gene expression events as a dynamic process and overcame the main limitations of bulk (ensemble) measurements: namely, the lack of subcellular resolution (nanometer to micron scale), the limited ability to detect subpopulations of cells, and the lack of insight into cell-to-cell heterogeneity. These three limitations have since been overcome, further highlighting the enormous complexity of transcription at the level of single cells. This complexity is perhaps also not so surprising, considering the central role of regulation of gene expression in the adaptation of cells to new conditions.

In this review, we provide an overview of the state-of-the-art imaging approaches to measure gene expression, from the early visualization of single genes by electron microscopy, to current single mRNA molecule and fluorescence-based approaches. To illustrate the advantage of the latest RNA imaging technologies, we discuss the coupling between transcription and translation in bacteria, from a biophysical and physiological perspective, with a particular focus on recent single-cell and single-RNA imaging studies. The coupling between transcription and splicing or between mRNA localization, translation and decay in eukaryotes has also been extensively studied using single-cell RNA imaging technologies; we refer the readers to specialized reviews on the topics [2,3,5].

## The ‘monochrome’ age of gene transcription visualization

**Figure 1.**
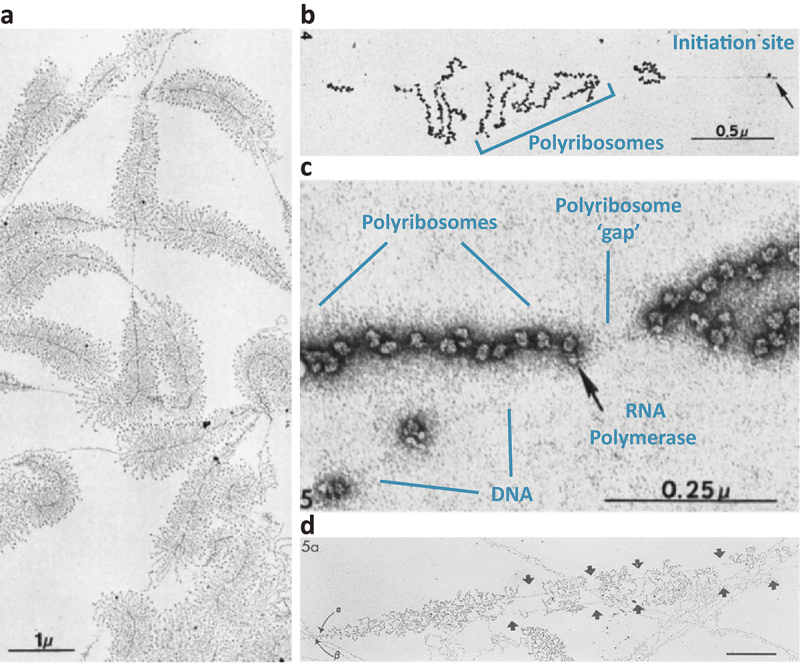
Electron micrographs of transcription units prepared by the Miller spreading technique. (a) Extrachromosomal rRNA transcription units from the newt oocyte (*Triturus Viridescens*) presenting the characteristic “Christmas tree” morphology. Virtually all rRNA units are fully loaded with transcribing RNA polymerases and separated by (non-transcribed) spacers. The “branches” of the trees are nascent rRNA chains which are progressively longer in the direction of transcription and decorated, at their end, by rRNA processing complexes appearing as “balls”. (b) An actively transcribed (and translated) bacterial operon showing the association of polyribosomes to nascent RNA chains. The position of the presumable transcription initiation site is indicated with an arrow. Note the irregular spacing between the ribosome-covered nascent RNAs, that Miller interpreted as discontinuity in transcription initiation. (c) A magnified view of an actively co-transcriptionally translated bacterial operon. A bacterial RNA polymerase is indicated with an arrow and it appears to directly interact with the leading ribosome. The variable length of the polyribosome “gaps” could indicate fluctuations in transcription initiation, now known as transcriptional bursting. (d) Miller spreads of Drosophila embryos showing paired post-replicative RNP fiber arrays containing internal transcript-free gaps indicated with arrowheads. The inferred transcription initiation sites are marked by α and β. (a-c) and (d) were adapted, with permission, from [13] and [14] respectively.

Prior to the widespread use of fluorescence microscopy, a series of landmark studies profoundly contributed to the early understanding of the spatial organization of transcription and the coupling of processes involved in the control of gene expression, in both eukaryotes and prokaryotes. The first visualization of genes caught in the act of transcription – *genes in flagrante transcriptio* [8] – were obtained by Oscar Miller and Barbara Beatty at the end of the 1960s by transmission electron microscopy, using a rapid and relatively nondestructive procedure [6]. This technique, which became known as a “Miller spread”, involved the dispersion of de-compacted chromatin fibers after cell lysis. The first genes observed were the newt oocyte ribosomal RNA (rRNA) genes. These resembled “Christmas trees” carrying about 80 to 100 RNA polymerases (later identified as RNA Pol I [9–12]), arranged as a train along a chromatin fiber giving rise to nascent rRNA precursors (Figure 1a). On these micrographs, the densely packed nascent ribonucleoprotein fibers (RNP) on each gene appeared progressively longer in the direction of transcription and were terminated by “balls”. These terminal knobs were later found to be rRNA processing complexes [15–17]. The precise localization of these iconic “Christmas trees” within the nucleolus was elucidated decades later with a combination of rDNA fluorescence *in situ* hybridization (FISH), BrU labeling of nascent pre-rRNA transcripts and immunolabeling, using both light and electron microscopy [18–20]. Because rRNA transcription units could be easily distinguished, Miller spreads were extensively used to study these genes in different cell types and during development, to explore questions that would have been difficult to address in cell populations. This technique was used, for instance, to uncover the organization and polarity of these genes, to provide evidence that rRNA genes can be differently regulated within an rDNA array, and that rRNA processing could occur co-transcriptionally [17,21–25].

Miller and coworkers also adapted their spreading technique to study bacterial transcription and they confirmed, visually, that translation could occur co-transcriptionally which was revealed by the association of polyribosomes to nascent transcripts (Figure 1b) [26]. Interestingly, the spacing between the polyribosomes along the chromosomes was found to be irregular (Figure 1c), which was interpreted as a possible discontinuity in transcription initiation. Fast-forward to 1999, Miller spread analyses of *Escherichia coli* cells, transformed with a multi-copy plasmid carrying an rRNA operon, revealed that the rate of rRNA expression per operon was reduced to maintain a constant concentration of rRNAs in the cell. Gene dosage compensation occurred without overall change in elongation rates, but via intermittent transcription or, what is now referred to as “bursts of transcription”, which the authors termed “gapping” [27]. These reports may constitute, respectively, the first indication of transcriptional bursting and one of the earliest attempts, at least in bacteria, to characterize this phenomenon now known to be widespread across all organisms [28,29]. Interrupted patterns of nascent transcripts were also observed in *Drosophila* embryos at multiple pairs of non-ribosomal genes on adjacent sister chromatids [14]. Intriguingly, in addition to displaying similar initiation frequencies, nascent transcripts-free gaps occurred at symmetrical positions on sister chromatid transcription units, suggesting some level of coordination between the initiation events at the two identical copies (Figure 1d). Because of the close proximity of sister chromatids, independently assessing the transcriptional activities of each copy of a replicated gene still remains challenging nowadays [30] with “classic” fluorescence-based live-imaging techniques (reviewed hereafter). The recent development of single-molecule nanoscopy [31] revealed that coordinated transcriptional bursting at sister chromatids in human embryonic stem cells was accompanied by an apparently shared pool of clustered regulatory factors, utilized by the two physically linked promoters [32].

Miller spreads were later used, in the 1980s and beyond, to visualize numerous processes associated with expression of RNA Pol II-dependent genes, including RNP structure, co-transcriptional splicing, and 3”-end processing [33–36]. Even the very short RNA Pol III-transcribed genes were visualized using this approach [37–39]. These early *ex-vivo* electron microscopy studies gave detailed visualizations (snapshots) of the initial events of gene expression. The genes investigated were limited to a restricted set that could be identified based on the already available biological information [6,37,38,40–42], by using cloned genes [36,43–45], or, more recently, by inserting genes of interest using the CRISPR/Cas9 technology near an easy to identify rRNA gene [39]. However, these experiments could neither assess the activity of the genes in their native context, nor track, in real time, dynamic changes in their expression, or the fate of the transcribed RNA after its release from the polymerase.

## Gene expression measurements in fixed cells: FISH-based methods

### Why measure mRNA expression *in* *situ*?

*In situ* Hybridization (ISH), is a technique that allows for the detection and localization of specific nucleic acids (DNA or RNA) within a genome, cell, or tissue. It was first described in 1969 for the localization of DNA sequences, such as rRNA genes on *Drosophila* polytene chromosomes [46,47]. Next, ISH was used in combination with isotopically- or biotin-labeled probes to investigate the localization of RNAs in single cells [48]. As opposed to chromatin spreads, this approach visualizes RNAs in morphologically intact cells, where the subcellular organization is preserved. This revealed that cytoplasmic mRNAs, such as the Actin mRNA in chicken fibroblast cells, are localized in nonrandom cellular locations where higher concentrations of mRNAs may result in the accumulation of the encoded protein [49]. A significant improvement of this method was achieved with the introduction of fluorescent probes combined to *in situ* hybridization (i.e., FISH), which provided increased resolution (from microns to ~250 nm), shorter protocol times (weeks to hours), and the possibility to perform multicolor imaging, as compared to isotope-based ISH [50]. The first RNA-FISH method showed that mRNAs within the nucleus were not restricted to the “Christmas trees” observed by Miller, but were instead found in areas that extended beyond the position of their cognate genes [50]. This provided a first hint of mRNA movement in the nucleus prior to their entry into the cytoplasm, highlighting the method’s potential in elucidating the spatiotemporal control of gene expression. Altogether, concomitant developments in the field of digital-image acquisition and fluorophore chemistry propelled the use of FISH as the reference method for the quantitative assessment of gene expression in single intact cells.

### State-of-the-art methods to measure mRNA molecules in cells

#### Fluorescence *in* *situ* hybridization: low-throughput approaches

**Figure 2.**
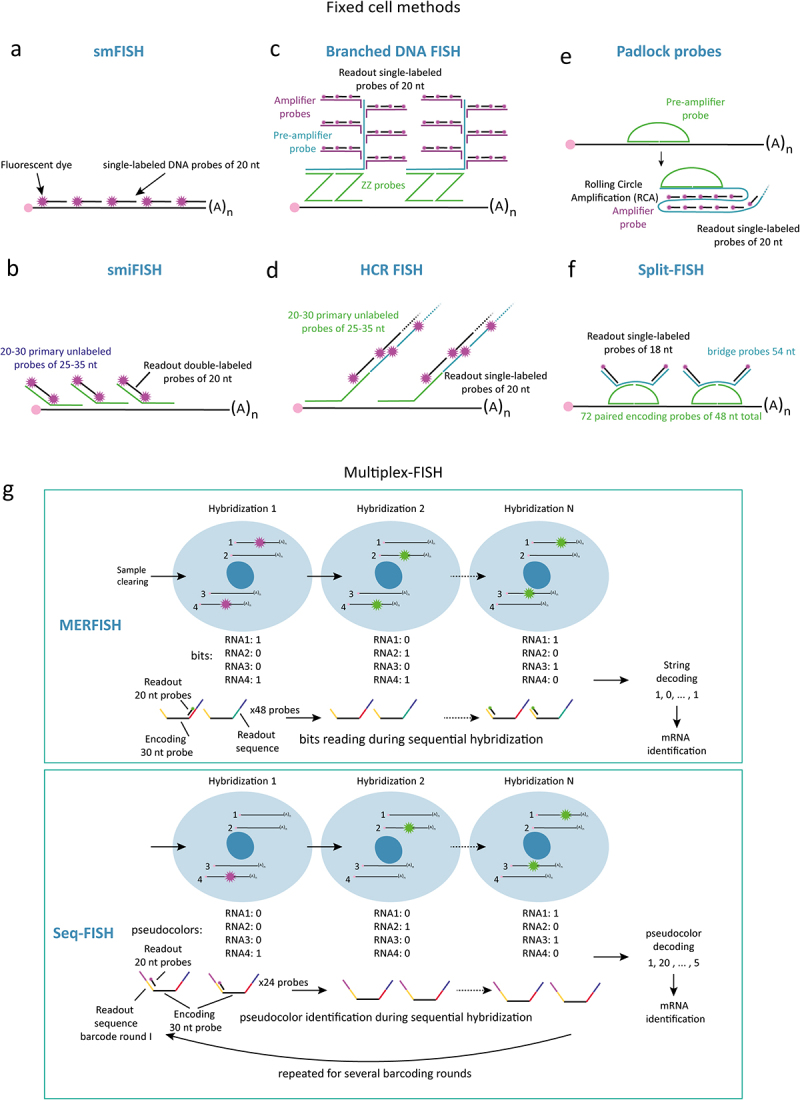
Overview of fixed cell FISH-based RNA imaging methods. (a) Schematic of standard smFISH probe design. smFISH signal amplification methods: (b) single molecule inexpensive FISH (smiFISH), (c) Branched amplification (bDNA, also called RNAscope). smFISH amplification via enzymatic reactions: (d) Hybridization chain reaction (HCR), (e) Padlock probes coupled to Rolling Circle Amplification (RCA). (f). Split-FISH is an example of probe design reducing nonspecific fluorescent probe labeling (g). Schematic of multiplexed smFISH methods: MERFISH and Seq-FISH have distinct barcoding schemes to encode mRNA. Seq-FISH uses several barcoding rounds, in which sequential rounds of hybridization are used to define a unique “pseudocolor” for each mRNA species. MERFISH uses a bit-barcode, encoded by several rounds of hybridization. In each round, a single mRNA transcript can be on (bit = 1) or off (bit = 0), creating a unique bit-barcode for each mRNA species.

Nowadays, FISH is generally performed by hybridizing an mRNA with 40 to 50 DNA probes of ~20 nucleotides, each singly labeled with a fluorescent dye (e.g., Quasars, Cyanine, CalFluors, Alexa; Figure 2a) [7,51]. Depending on the microscopy filter setup, simultaneous detection of 1 up to 4 distinct mRNA species per cell, or the simultaneous detection of mRNA and proteins by immunofluorescence, has now become standard [52–56]. In combination with fluorescence microscopy (e.g., wide-field, confocal) this method is now sensitive enough to detect single mRNA molecules (i.e., smFISH), allowing for their counting, across a wide range of organisms, from bacteria to entire multicellular eukaryotic organisms [2,57]. Even when compared to single-cell sequencing approaches, smFISH excels in the detection of lowly expressed mRNAs [58,59]. This is particularly useful for the study of gene expression in microbial species, where cells synthesize, per gene, less than ten mRNA molecules per cell on average [60–62]. For the detection of mRNA molecules in samples with high autofluorescence (e.g., tissues or cells with a thick cell wall), or of short mRNAs (i.e., <500 nucleotides), various fluorescence amplification strategies have been developed. Methods such as smiFISH [63] and Branched DNA FISH [64] rely on binding to the target mRNA of a primary non-fluorescent probe with an overhanging tail that is hybridized either directly by a fluorescent readout probe, or indirectly via a secondary amplifier probe (Figure 2b-c). Additional amplification can be achieved with enzymatic approaches such as Hybridization Chain-Reaction-FISH (HCR-FISH) [65,66] or Padlock probes coupled to *in situ* Rolling Circle Amplification (RCA) [67,68] (Figure 2d-e). A potential downside, however, is that the high degree of amplification comes at the cost of the robustness of mRNA spot intensities and, therewith, the precision of mRNA counting.

#### Fluorescence *in* *situ* hybridization:high-throughput approaches

Rapid advances have been made in multiplexing smFISH in the last ten years. In multiplexed smFISH methods, optical barcodes are used to detect and localize tens to thousands of mRNA species simultaneously. Early multiplexed methods generated unique patterns of fluorescence by hybridizing a specific mRNA with probes with different combinations of fluorophores [69–71]. Later methods, improved this design by encoding the fluorescence-based barcoded signal across multiple rounds of hybridization, imaging and signal removal [52]. The two most widely used methods are Sequential FISH (SeqFISH) [54] and Multiplexed error robust FISH (MERFISH) [53]. For an in-depth analysis of the latest mRNA and protein spatial profiling methods, we refer the readers to a recent extended review [72]. In the next paragraph, we summarize the key characteristics of multiplexed FISH methods (Figure 2g).

The proof of concept that optical barcodes can be generated by sequential rounds of hybridization was first published by the SeqFISH team [52]. This initial design allowed for the identification of 12 mRNA species in *S. cerevisiae*. By using four different fluorophores, and two rounds of hybridization, they generated 4^2^ = 16 unique color combinations. Each mRNA was hybridized with probes labeled with one fluorophore, imaged, after which the probes were removed by digestion and photobleaching. Next, a second round using probes labeled with another fluorophore was hybridized and imaged. In this way, each mRNA species could be assigned a unique optical barcode. To improve the initial design, SeqFISH used a multiplexing approach based on designing a high number of readout probes. For instance, to detect ~10,000 mRNAs, 240 unique readout probes were designed. For each mRNA, 28–32 encoding probes were synthesized, each fused to 4 out of the 240 readout probes. The detection relied on 80 sequential hybridization rounds and 3 fluorophores [54]. This allowed to perform spatial transcriptomics on mouse brain tissue – although with an estimated 50% detection efficiency [54].

Next, the MERFISH team developed a new encoding approach to reduce error accumulation caused by the high number of hybridization rounds and mRNA density. In MERFISH, to detect 10,015 mRNAs, 69 distinct readout sequences were used. These readout sequences were decoded by 23 rounds of sequential hybridization and fluorescent probes labeled in 3 colors. To prevent errors in mRNA decoding, the optical barcodes of MERFISH are designed according to the telecommunication concept of hamming distance, an encoding strategy that ensures that individual barcodes are at least separated by a certain number of misclassified bits (4 in MERFISH). Remarkably, the MERFISH method was applied to measure mRNA expression levels of 10,000 genes in human osteosarcoma cells with up to 80% detection efficiency [73]. To further improve signal-to-noise ratios, SeqFISH and MERFISH have been combined to strategies to increase signal-to-noise ratios. For instance, sample embedding in transparent gel matrices and clearing were applied to reduce cellular background fluorescence in human fibroblast cells and mice brain tissue [54,74]. Alternatively, multiplexed FISH has been coupled to amplification techniques such as branched amplification, HCR and RCA [68,74–77] (Figure 2c-e).

To limit nonspecific binding of probes to off-target mRNAs, different groups have produced sophisticated probe designs [78–81]. In Split-FISH, for example, the binding of adjacent probes is required for the binding of a fluorescent readout probe, diminishing nonspecific fluorescence probe labeling (Figure 2f). Furthermore, high transcript-density areas mask the fluorescence of neighboring transcripts. This can be improved by either increasing the sample size, as seen in FISH-expansion [82], or by distributing the fluorescent signal over more rounds of hybridization and by increasing the number of readout probes. These improvements in probe specificity and signal-to-noise ratio did not only help correct barcode decoding but also enabled the imaging of mRNAs in tissues and organisms with thick cell walls, where the probe diffusion efficiency may be lower and autofluorescence is higher. Together, MERFISH and SeqFISH have been used to profile the transcriptomes in mammalian cells and tissues (e.g., brain, fetal liver, gut [72]), and also the expression of genes in pathogenic microbes [83].

## Gene expression dynamics: RNA measurements in living cells

### Why measure mRNA expression in living cells?

While fixed cell methods are highly quantitative and allow for the simultaneous detection of up to thousands of mRNA species in single cells, they do not capture the dynamic changes in gene expression occurring in living cells. This is important when investigating cell adaptation to environmental changes occurring on a time scale of minutes and involving rapid integration of signaling and metabolic changes which feedback to the gene expression machinery.

Furthermore, given the short time scales of molecular diffusion in living cells, real-time gene expression reporters can provide insights on the dynamic molecular regulation of gene expression and in turn cell physiology. In his seminal 1828 paper, Robert Brown reported the first careful account of particles movement in plant pollens [84]. This study was followed by numerous measurements of “Brownian” motion of biomolecules in living cells [85]. For instance, in *E. coli*, tRNAs have been reported to have apparent diffusion kinetics ranging from 0.5 to 20 μm^2^/s [86], while proteins, depending on their size, have diffusion coefficients ranging from 1 to 7 μ m^2^/s [87,88]. Mobility of mRNAs in eukaryotic cells varies widely and depends on whether an mRNA is freely mobile (apparent diffusion kinetics of 0.1–0.4 μm^2^/s [89]), or if they are actively transported within a cell (speed close to 0.5–2 μm/sec) [90,91]. Altogether, these measurements underscore the “need-for-speed” that stirred the development of live single-cell gene expression reporters.

Advancements in the field of biophysics have significantly contributed to recent technological leaps. These include the development of high-speed sensitive detectors [92] and of fluorescent molecules, i.e., fluorescent proteins (FPs) and dyes, with improved brightness and photostability [93–96]. Technological progress has also been accompanied by the improved availability of user-friendly customizable microscopy setups and the standardization of microscopy practices [97], which have all contributed to the increasingly widespread use of these technologies.

### State-of-the-art methods to measure mRNA molecules in living cells

Current mRNA-imaging methods can be divided into two major groups: those that require the genetic modification of the target mRNA with an aptamer reporter and those that target the endogenous mRNA directly.

#### Aptamer-based reporters

**Figure 3.**
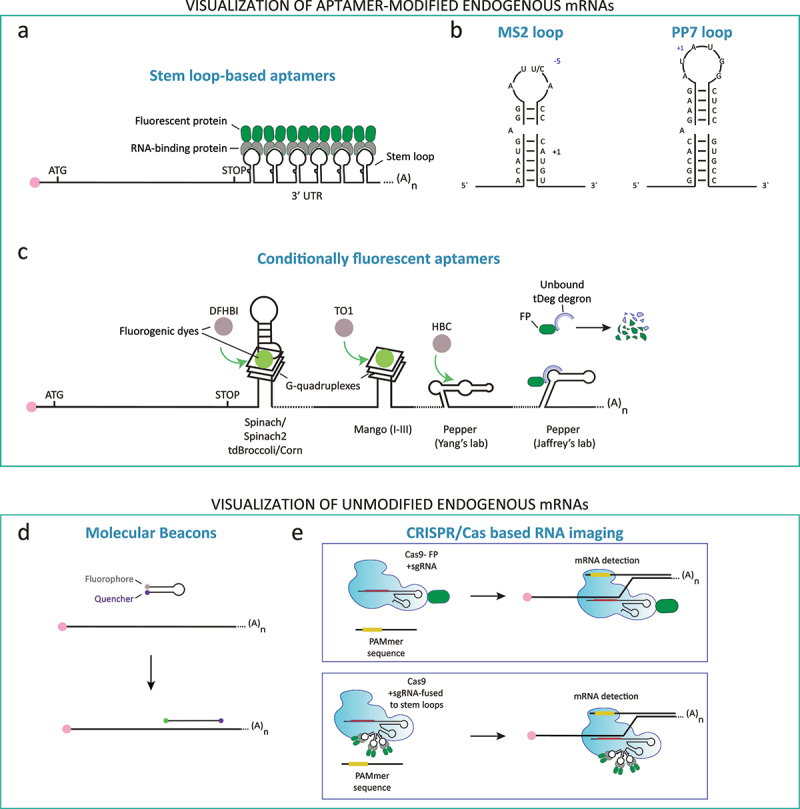
Methods to visualize and measure mRNA molecules in living cells. Fluorescence-based mRNAs visualization methods rely on either aptamer-based modification of the endogenous mRNA (Top box), or by detecting endogenous unmodified mRNAs (Bottom box). (a) Aptamer-based reporters include stem loop-based systems, whereby arrays of loops can be integrated in the 3’ UTR (or 5”UTR) of a gene of interest. The loops are bound by specific RNA binding proteins which are fused to fluorescent proteins. (b) Single-molecule mRNA detection frequently relies on repetitions (12 to 24) of MS2 or PP7 loops. Another class of aptamer-based reporters are conditionally fluorescent systems. (c) to visualize single mRNAs an array of RNA structures such as Spinach/2, tdBroccoli, Corn, Mango(I-III), Pepper (Yang’s lab) can be inserted in the 3”UTR of the mRNA of interest. Fluorogenic dyes become fluorescent upon binding to the RNA aptamers. Another fluorogenic system, was recently developed by the Jaffrey’s lab, also called Pepper, which relies on an RNA loop-regulated fluorescent protein degron-system. (d) Visualization of endogenous unmodified genes can be achieved by molecular beacons, or (e) by CRISPR – Cas systems: Cas9-GFP, Cas9-MS2 or PP7-fusion or dCas13-EGFP to target a specific mRNA via single guide RNAs (sgRNAs).

Aptamer-based mRNA imaging approaches rely on the expression of an mRNA joined either to RNA stem-loops (Figure 3a) or directly to fluorogenic – i.e., conditionally fluorescent – aptamers [98–104]. The insertion of 24 loops in the gene of interest is usually sufficient to allow single-molecule RNA visualization, even though the use of 96 or 128 loops have been reported for further signal amplification [105,106]. When localizing mRNAs in living cells, loops are generally inserted in the 3’ untranslated region (UTR) of a gene, immediately after the stop codon [91,99,101,107]. On the other hand, to measure transcription dynamics, stem-loops are inserted either in the 5’ UTR, before the first translation initiation codon, or within introns, which provides a rapid readout for RNA synthesis, since the nascent RNAs become fluorescent as soon as they emerge from the elongating RNA polymerases. Caution is required when inserting stem loops in 5’ UTR of an mRNA, since the stem loops, bound by the cognate-binding proteins, display a well-characterized translation inhibition function [108–110]. Thus, tagging of essential genes with loops may in itself cause aberrant cellular phenotypes [107,111–115]. Over the past three decades, numerous stem-loops have been optimized for aptamer-based mRNA imaging. They are derived either from bacteriophages (e.g., MS2 [107], PP7 [116,117], P22 [118]) or from RNA-protein binding pairs (e.g., U1A [119,120], Bgl bacterial anti-terminator [121], Pumilio recognition motif [122]). Association of stem-loop arrays with cognate RNA binding proteins (RBP) fused to an FP (e.g., GFP) or a fluorogenic tagging system (e.g., HALO) allows detecting individual mRNAs in living cells. The best characterized and most widely employed aptamer loops are MS2 and PP7. Their popularity is likely due to their reliable detection of single mRNA molecules across a wide variety of living cells with minimal perturbation of cell physiology. The binding of the MS2 and PP7 loops to the cognate coat protein has been structurally characterized in great details [123], prompting the development of improved variants. For instance, the first MS2 arrays developed for RNA imaging took advantage of a point mutation in the MS2 loop at position−5 relative to the translation start site (Figure 3b), which converted a uracil into a cytidine, increasing by tenfold the affinity of the MS2 coat protein (MCP), from a K_d_ of 10 to 1 nM, and slowing the dissociation kinetics by about 90 times (minutes to hours) [124–126]. This increase in affinity was aimed at improving the detection of single mRNA molecules in living cells. However, the downside of such high-affinity reporters was their inefficient degradation when used to tag mRNAs in *S. cerevisiae* [99,112,127,128], where mRNAs have short half-lives (median average of 20 min [129]). New MS2 variants, with optimized affinities, distance between loops, optimized MCP expression levels and the use of non-repetitive loop and linker sequences, resulted in a flexible toolbox for mRNA visualization optimization [57,99,100,106,130,131].

MS2 and PP7 have been used to visualize single mRNA molecules in bacteria (e.g. [106,132–135]), fungi (e.g. [99,101,107,117,136–140]), amoebas (e.g. [141,142]), plants [143–145], insects (e.g. [98,113,114,146,147]), frog [148], fish [149] and mammalian cells (e.g. [102,150–153]). The MS2 and PP7 tagging systems are orthogonal since they share only 15% sequence identity allowing for specific recognition by different cognate-binding proteins, MCP and PP7 coat protein (PCP), respectively [116] (Figure 3b). This enabled the combined use of the two systems to simultaneously tag, within the same cell, either two mRNA species or the same mRNA at multiple sites [136,154–156]. To visualize mRNAs in living cells, MCP or PCP, fused to FPs or fluorogenic tags (e.g., eGFP, mCherry, HALO) are generally expressed from constitutive low-expressing promoters [57,99,104,157]. This ensures that as soon as a mRNA is transcribed, it becomes fluorescent upon coat protein binding. The disadvantage of having constant expression of the fluorescent coat protein is the potential high background fluorescence. To counter this limitation in mammalian [158] and plant [159] cells, an aptamer version was developed, whereby hybrid arrays alternating MS2 and PP7 loops are recognized by MCP and PCP proteins each fused to a split-GFP domain. A further disadvantage of the MS2 and PP7 systems is the bulky size of the aptamer bound by the FP-fused coat proteins, which can reach a mass of about 2–3 megadaltons thus possibly affecting mRNA diffusion and transport dynamics.

To overcome the shortcomings of the MS2 and PP7 system, namely its bulkiness and background fluorescence, a class of fluorogenic aptamer-based reporters was developed to improve RNA detection in living cells. These aptamers use a soluble form of the GFP fluorophore, which is minimally fluorescent in solution and fluoresces upon binding to an RNA aptamer [160] (Figure 3c). Examples of aptamers include: Spinach [160], Spinach2 [161], Broccoli [162], Corn [163], Mango [164–166], DNB and SRB-2 for *E. coli* [167]. While all these aptamers have excellent fluorogenicity, they do not allow for the detection of single mRNA molecules because of their lesser brightness compared to eGFP [167,168]. A new variant of Mango, dubbed Mango-III-A10U, showed promising results *in vitro*. This aptamer is brighter than eGFP and with an aptamer-fluorophore dissociation constant in the nM range [169]. Moreover, the development of a multicolor riboswitch-based RNA imaging system, called Riboglow, was recently reported [170,171]. This reporter exhibited twice the brightness of eGFP with a dissociation constant in the nM range. When used for RNA imaging in live mammalian cells, Riboglow performed similarly to the gold-standard MS2 system [170,171]. Altogether, these fluorogenic aptamer-based reporters showed the most rapid development and the greatest improvement over the past decades, but their use is still limited in the field of cell biology. This is possibly due to the fact that not all the fluorogenic fluorophores are cell permeable and, in some cases, bead-loading is required for fluorophore uptake [170]. Furthermore, like for the MS2 system, single-molecule imaging capability is only possible when arrays of fluorogenic aptamers are used to tag the mRNA of interest [172,173]. Thus, only abundant endogenous RNAs have been visualized in living cells [160,161,163]. The effect of the insertion of these aptamers onto the target mRNA expression (i.e., translation and stability) has also not been systematically tested. This requires particular attention, since the above mentioned fluorogenic aptamers contain G-quadruplexes structures, which can influence various steps of gene expression [174,175]. Promisingly, a new G-quadruplexes-free fluorogenic aptamer, Pepper, was recently developed by the Yi Yang’s group [176], possibly paving the way for the development of minimally perturbing fluorogenic mRNA tagging systems.

In the same year, the Jaffrey group described the development of another aptamer, also called Pepper [177]. This aptamer does not bind a soluble fluorophore, like the other “vegetables” aptamers, but it uses an innovative approach to generate a fluorogenic RNA detection system. Jaffrey’s lab Pepper is *de facto* a protein degron system, as it involves the Pepper RNA aptamer binding to the bifunctional peptide “tDeg”, which contains a degron sequence, thereby preventing its degradation (Figure 3c). Conversely, in absence of Pepper the tDeg protein is rapidly degraded. This system was used to generate tDeg-FP fusions that allowed the visualization of mRNAs tagged with Pepper arrays in living mammalian cells [177]. The limitation of this approach is that the Pepper-tagged mRNA needs to be stable enough to allow binding by the tDeg-FP fusion and maturation of the FP, which is required for efficient mRNA detection. Thus, this approach may be not optimal to measure the expression of unstable mRNAs.

The newest member of the aptamer-based mRNA visualization toolbox takes advantage of the CRISPR-Cas system. Either Cas9 or Cas13 fused to FPs have been used for RNA imaging in living cells upon modification of the target mRNA with aptamer sequences for signal amplification [178–180]. Interestingly, the use of fluorophore-conjugated guide RNAs for detection [180], instead of FPs fused to the Cas proteins, may promote the development of multicolor detection systems, since synthetic fluorophores have usually smaller sizes and they can be modified to produce a vast range of fluorescence spectra [181].

#### RNA imaging approaches targeting unmodified endogenous RNAs

Modification of a target RNA with an aptamer can have significant consequences for its expression. To overcome the limitation of aptamer-based reporters, two live-cell imaging reporters have been described: Molecular Beacons (MBs) and a CRISPR/Cas-based RNA imaging systems (Figure 3d).

MBs are oligonucleotides conjugated at their 3’ and 5’ end with a fluorophore and a quencher, respectively, and they are introduced in cells by electroporation [182–184]. When the MB is not hybridized to the target mRNA, the fluorophore is inactivated by the quencher; this dark state is reversed upon MB binding to the mRNA. In a recent report, it was shown that MBs, in combination with highly inclined and laminated optical sheet microscopy, afford single-molecule mRNA detection in live *Xenopus* retinal ganglion cell axons [185]. More studies are needed however, to validate the use of this RNA-imaging approach.

Lastly, it was shown that the CRISPR/Cas system can be used to visualize endogenous, unmodified RNAs via single guide RNAs (sgRNAs) targeting Cas9-GFP to specific transcripts [186,187]. Furthermore, the CRISPR/Cas9 system was combined with aptamer-based signal amplification [188], whereby sgRNA fused to arrays of MS2 or PP7 loops were used for multicolor RNA detection upon binding of MCP-EYFP or PCP-tagRFP, respectively. Even though these pioneer studies did not yet allow single-molecule RNA detection, they represent a promising alternative to aptamer-based systems.

## Investigating gene expression coupling in bacteria

**Figure 4.**
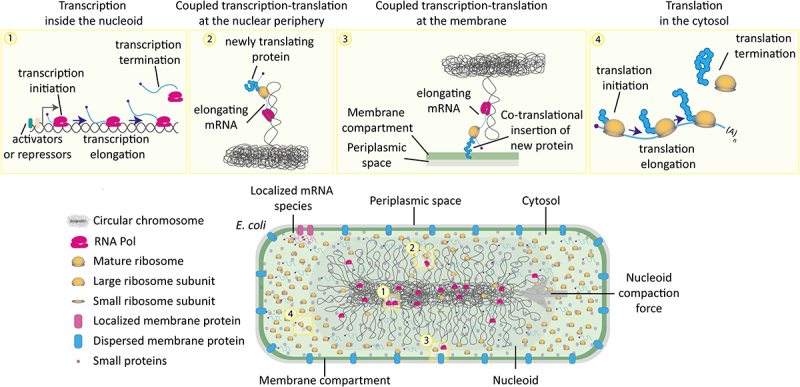
Spatial and molecular mechanistic aspects of transcription and translation in *Escherichia coli*. *E.* *coli*’s transcription and translation occur in a crowded cytoplasm with a compacted nucleoid, a too-thin DNA mesh for assembled ribosomes to diffuse through, but RNA polymerases and other smaller proteins can diffuse through. Ribosomes therefore predominantly reside excluded from the nucleoid while RNA polymerases do not. Both are mostly bound to their substrates, i.e., mRNA and DNA, and RNA Pol remarkably often to unspecific DNA sites, unrelated to transcription. Due to the localization of ribosomes, translation is expected to occur mostly outside of the nucleoid and at its periphery it can occur coupled to transcription and insertion. Most mRNA are therefore also found outside of the nucleoid, and assist in its compaction, and reside most of their lifetimes as finished transcripts, only 6% of their life time are they being transcribed; indicating that coupled transcription and translation may serve regulatory function instead of contributing to the enhancement of protein yield per transcript.

Taking advantage of high-resolution protein and mRNA imaging technologies, recent studies revealed that, in bacteria, RNA polymerases, ribosomes and many transcripts are localized in a nonrandom fashion [88,189,190], challenging the once long-held view that bacteria are “unorganized bags of molecules” (Figure 4). In addition, processes involved in gene expression such as transcription and translation have been themselves shown to be effectively compartmentalized in multiple bacterial species [88,191]. In the next paragraphs, we summarized quantitative consideration regarding bacterial mRNA transcription, localization and the coupling of transcription with translation, in light of recent single molecule and single-cell gene expression studies.

### Quantitative biology of bacterial transcription: general considerations

When we limit ourselves to balanced growth of cells [192,193], the concentration, synthesis rates and degradation rates of proteins and mRNAs are at steady state and are therefore quantitatively related to each other. The rate of protein synthesis depends on the mRNA concentration (amongst other factors) and needs to balance its degradation rate and dilution, due to cell-volume growth. The balancing of these rates determines the required mRNA concentration, given its degradation rate, which equals its synthesis rate at steady state. This sets the required transcription activity of the gene, which equals its transcription initiation rate at steady state. The initiation rate is then determined by the type of promoter (i.e., its DNA sequence), the local DNA structure and concentrations of RNA polymerases and regulators. The total cellular biosynthetic resources for transcription and translation, concentrations of RNA polymerases and ribosomes are growth-rate dependent [194], these codetermine the initiation rates of transcription and translation. Thus, a quantitative view of transcription needs to take cellular growth rates into consideration [195]. Finally, the number of active genes partially determines the free concentration of RNA polymerases, affecting global transcriptional rates [194,195].

#### Transcription activity

In *E. coli*, between 1,500 and 5.000 RNA polymerases, about 96% of the total polymerases, are found in the nucleoid – an irregularly shaped globular structure that contains the circular chromosome of *E. coli* [196] -, suggesting that they are mostly engaged in transcription [197]. The first study, published on the localization and timing of mRNA synthesis in living bacterial cells, took advantage of the MS2 tagging system [106]. An array of 96 MS2 loops was cloned on an expression plasmid, which allowed the visualization and quantification of bright transcription sites associated with the DNA. In a follow-up study from the same group, the MS2 reporter allowed to investigate the real-time kinetics of *E. coli* transcription, revealing that it occurs in quantal bursts [135], as previously suggested based on electron micrographs of Miller spreads [14,26,27]. Thanks to their ability to count absolute mRNA numbers in *E. coli* cells, Golding and coworkers demonstrated that each transcriptional burst is characterized by a specific size, which is geometrically distributed [135]. Thus, transcripts are produced in batches during intense ON periods of transcription, while they are degraded during the OFF periods. Furthermore, they showed that transcription in *E. coli* is infrequent, and that the time intervals between bursts are exponentially distributed [135], consistent with a simple gene activation-inactivation model whereby the association of low-copy transcription factors and RNA polymerases to gene promoters triggers transcription activation [135]. Additional single-cell studies using smFISH investigated the effect of different promoters on the mRNA copy numbers in bacterial cells. This revealed that the promoter activity is generally non-bursty for low-expressing promoters (less or equal to 1 mRNA/cell), while burstiness increases with gene activity [198]. In addition, it was shown that mRNA counts per cell correlate better with the duration of each burst, which is mainly set by the gene OFF state, rather than the burst size. Thus, the number of bursts matters more than the number of mRNAs made per burst [198]. The switching between the ON and OFF states and mRNA expression heterogeneity has been elegantly associated to cellular phenotypes, such as the ability of *B. subtilis* cells to uptake DNA [199] or the *E. coli* response to the bacteriophage lambda infection [200]. A comprehensive review on the topic of gene expression noise in bacteria was recently published [201].

#### The localization of mRNAs in *E.* *coli*

The once held view that bacteria resemble unorganized bags of macromolecules has been challenged by studies showing that many transcripts localize in *E. coli* where their protein products are needed [132,133,202]. In these cases, the mRNA likely carries the localization signal, and the protein is subsequently made on site, as soon as the mRNA has been delivered [189,203]. RNA imaging technologies such as the MS2 system and smFISH did not only allow to elucidate fundamental aspects of transcription but also where mRNAs are localized in bacterial cells. Few studies systematically investigated subcellular mRNA localization in *E. coli*, but they revealed a surprising degree of spatial organization [132,133,189,202]. By using a combination of MS2 system and smFISH, a seminal paper from the Amster-Choder group showed that certain mRNAs are targeted where their encoded proteins are localized: in the cytoplasm, the poles, or at the inner membrane [133]. This observation was further corroborated, across a wide variety of bacterial species, by single-gene studies [200,204–208], multiplexed RNA imaging for RNA clusters (i.e., multiple functionally related targets) [202] and genome-wide approaches [132], implying that most mRNAs are not associated with the nucleoid, as previously suggested for *C. crescentus* and *E. coli* [209]. While the mechanisms controlling asymmetric mRNA distribution in bacteria are not fully elucidated, few clues are currently available. Several possible scenarios can be envisaged based on the extensive characterization of mRNA localization in eukaryotic cells (reviewed in [2]). RNAs could be targeted to subcellular compartments thanks to so-called RNA “zip-codes”, which are secondary structures found in an RNA sequence and recognized by RBPs actively targeting RNAs within a cell via motor proteins. Alternatively, freely diffusing mRNAs could be asymmetrically distributed thanks to the specific binding to anchored RBPs or, indirectly, via the subcellular localization of RNA degrading enzymes. Finally, mRNA localization could be coupled to translation of protein localization signals (e.g., protein inserted in the bacterial membrane) (Figure 4).

Recent studies suggest that most RNAs in *E. coli* are accumulating at their final destination in a translation-independent manner [132]. Nonetheless, it was shown that mRNAs encoding trans-membrane proteins are addressed via signal sequences recognized by signal-recognition particle (SRP) in a translation-dependent manner [202]. Furthermore, it was shown that the degradation rates of inner-membrane-protein mRNAs are on average faster than those of the other mRNAs [202], consistent with previous studies showing that the *E. coli* RNA degradation machinery (RNAse E) is associated with the membrane [4,210]. Finally, it was demonstrated that stress-related mRNAs and small RNAs (sRNAs) are enriched at the bacteria poles [132]. Interestingly, sRNA localization at the poles is stimulated upon stress and it depends on the RNA chaperone Hfq [132], a key factor promoting the pairing of sRNAs to target mRNAs, regulating mRNA stability and translation [211]. Further studies will need to elucidate the functions of subcellular localization of RNAs in bacteria, possibly adding a layer of regulation important to control not only protein localization but also mRNA stability and in turn protein expression.

### Macromolecular crowding and emergent compartmentalization of transcription and translation

The spatial organization of macromolecules in the bacterial cell is influenced by both macromolecular crowding [212,213] and by entropic (depletion) forces which compact the nucleoid and expel bulky, translating ribosomes. In contrast, RNA polymerases predominantly reside in the nucleoid, while small proteins (e.g., metabolic enzymes) spread homogeneously in the cytosol [88,197]. As a result, most mRNAs are expected to be outside of the nucleoid [214], which was confirmed by the RNA localization studies discussed in the previous paragraph. In turn, the macromolecular composition of the bacterial cytoplasm influences rates and spatial organization of transcriptional and translational processes [88,201,215]. In the next paragraphs, we discuss how a quantitative understanding of cellular compartmentalization can help elucidate the extent and function of transcription-translation coupling in bacteria.

#### The structure of the nucleoid

The nucleoid in *E. coli*, which consists of a DNA molecule of ~1.5 mm in length, is compacted in a single cell about 2–4 µm long, by over a 1000-fold when compared with its linear length, thanks to the presence of crowding forces [216]. Hence, the nucleoid compacts to a size smaller than the cell and positions itself in its center [88,217]. Nucleoid compaction is found in many bacteria, although to different degrees [191]. The extent of compaction is dependent on transcriptional activity as its inhibition with rifampicin causes nucleoid expansion [218]. In addition, during slow growth, the nucleoid is more spread throughout the cytosol than at fast growth [219]. Due to a fixed DNA replication time, *E. coli* may contain multiple replicating copies of DNA during fast growth when the generation time is shorter than the DNA replication time. This is also accompanied by a change in the nucleoid size, occurring, remarkably, at a constant DNA over protein ratio (called the unit cell) [220].

The nucleoid compaction is associated with the nucleoid exclusion of large macromolecular complexes – such as aggregates of misfolded proteins and assembled, translating ribosomes – while small proteins randomly disperse [88,221]. Ribosomes are bulky complexes with diameters of 20 nm relative to other proteins, with an average diameter of 5 nm. Since about 80% of ribosomes are actively translating [194], the maximal fraction of unassembled ribosome subunits is 20% and those are small enough to homogeneously spread through the cell and enter the nucleoid as smaller proteins and complexes do [215]. About 80% of all RNA polymerases reside in the nucleoid attached to DNA, with about 60% of them nonspecifically bound to DNA, while about 20% are actively transcribing, and the remaining 20% are not bound to DNA (assembled and partially assembled) [195]. Unbound RNA polymerases can explore the entire nucleoid, in contrast to assembled ribosomes, and engage, for 85% of their promoter search time, in unspecific DNA interactions [222], which is why they nearly all reside in the nucleoid [197].

#### Why so crowded?

Given all the complexities associated with macromolecular crowding, why is the interior of cells so crowded with macromolecules? One plausible explanation comes from a fitness consideration: the fastest growing cells win the evolutionary competition, they make copies of themselves at the fastest rate and, therefore, have the highest rate of metabolism and biosynthesis. Since those rates are each proportional to the concentration of their catalyzing enzyme, these concentrations should be as high as possible. However, when they are too high, they enhance the cellular viscosity too much, and reduce the diffusion rate, which would reduce growth rate (the diffusion coefficient *D* is inversely proportional to the viscosity of the medium). Thus, an optimal protein density exists, which represents the optimal trade-off between those two-opposing forces, and maximizes growth rate. *E. coli* appears to be close to this value [197]. It has been suggested that the rate of translation is limited by the diffusional supply of loaded tRNAs [197], indicating the importance of diffusion inside cells.

Due to macromolecular crowding, the diffusion coefficient of a protein, like GFP, is about 11 times slower in an *E. coli* cell than in water (D = ~8 $\mu m^{2}/s$), and about 3-fold slower than in a eukaryotic cell [197]. This implies that a molecule travels the length of an *E. coli* cell (2 µm) in about 0.1 s. The collision time for two proteins equals $\frac{0.6}{N_{1}N_{2}}s$, in a 1 femtoliter cell and at $N_{1}$ and $N_{2}$ copies per cell. At a growth rate of 1 $hr^{-1}$, the free concentration of RNA polymerases in *E. coli* has been estimated to be about 400 molecules per cell [195], which means that, on average, every 0.0015 s (0.6/400 s) an RNA Pol collides with a promoter, leading to association or an unproductive collision. Whereas a transcription factor occurring at 10 copies per cell would take about 0.06 s. For quantitative measurements of the search time that a transcription factor needs to find its target, see Elf et al. [223] who reported a search time of ~270s for the *lac* repressor, which is so slow due to many unproductive collisions.

Bakshi et al. found that ~ 85% of the ribosomes reside outside of the nucleoid and they suggested that therefore only ~15% would be engaged in coupled transcription and translation, since the gene density at the periphery of the nucleoid is unknown, this may be an underestimation (likely not severely so) [197]. They concluded that the 30S subunit of the ribosome (containing: a 1540 nucleotide-long 16S rRNA and 21 proteins) has a diffusion coefficient of about $0.14\mu m^{2}/s$ (about 50 times slower than GFP) and the actively-translating assembled ribosome has a diffusion coefficient of about $0.02\mu m^{2}/s$. Thus, actively translating ribosomes, which are essentially all outside of the nucleoid, hardly move and exist in a mRNA-rich compartment (i.e., mostly the cell poles at low and intermediate growth rates), where they are also likely “caged” as they will quickly encounter a new mRNA upon finishing translation [197].

#### Quantitative analysis of transcription and coupled transcription-translation

In contrast to the early report by Miller that essentially all polyribosomes were connected to the *E. coli* genome [26], more recent work suggests that the coupling between transcription and translation does not apply to all genes [224]. Interestingly, the local structure of the nucleoid, and therefore the location of genes in it, appears to be influenced by the rate of transcription. Actively transcribed genes preferentially cluster at the periphery of the nucleoid, while low transcriptional activity occurs throughout the nucleoid [222], such that active genes produce transcripts that are directly exposed to ribosomes, favoring their coupled transcription and translation. Thus, a combination of physical forces and genetically encoded regulation of gene expression likely determine the probability of transcription-translation coupling.

Since the average transcription time for a gene is about 20s and the average life time of an mRNA is 5 min [60], coupled transcription and translation can occur for only about 6% of the mRNA lifetime and, likewise, about 6% of all proteins made from this mRNA can be made via transcription and translational coupling. Moreover, whether coupled transcription and translation can occur depends on how accessible the mRNA is for ribosomes. This appears to be the case only for genes on the periphery of the nucleoid [222]. Since only 6% of the proteins are made during coupled transcription and translation, this does not greatly affect the protein pool. Furthermore, considering that the transcription time is only 6% of the lifetime, the benefit of transcription and translation is also really not a matter of the speed to make the first transcript. Without coupled transcription and translation, the synthesis of the first protein would thus be delayed by only 20 s plus the translation initiation time for the protein. However, even if protein synthesis rates are likely not directly dependent on aspects of coupled transcription and translation, they could still be dramatically affected by the coupling of these processes, if productive transcription at particular genes depends on it [225–227].

## Closing remarks and future challenges

Sydney Brenner famously said “I will ask you to mark again that rather typical feature of the development of our subject; how so much progress depends on the interplay of techniques, discoveries and new ideas, probably in that order of decreasing importance” [228]. A succession of technological breakthroughs, from the Miller spreading technique to the development of super-resolution fluorescence microscopy, have enabled an ever-increasingly detailed visualization of genes “in action”. Observations made along the way revealed, or confirmed, the complexity and the interdependency of the processes involved in the expression of a gene. Genetics, biochemistry and later molecular biology approaches, which mainly relied on ensemble measurements, have undoubtedly driven the advancement of our understanding of gene expression for decades, often pointing out where to look, what to image or sometimes even what was being observed. In turn, electron and later fluorescence microscopy revealed the organization and spatiotemporal control of these genes, and provided the tools to evaluate the cell-to-cell variability in their expression as well as to track, dynamically, the fate of their RNA products.

The toolbox of RNA imaging reporters is continuously expanding, enabling finer investigations of the spatiotemporal control of gene expression, but several challenges still need to be tackled. For instance, RNA imaging in fixed cells (i.e., using smFISH-based approaches) can now be applied at the scale of the whole transcriptome. This brought interesting new challenges such as the need for automated imaging analysis algorithms capable of outlining thousands of cells with diverse morphologies. Moreover, *in situ* transcriptomic protocols still remain to be adapted for their use in single or multicellular microbial organisms. This could notably benefit studies aiming at understanding interactions between pathogenic or industrially relevant species and their environment. On the other hand, RNA imaging in living cells still requires further improvements, to further reduce the impact of tagging RNA of interests with bulky arrays that may affect their metabolism. Furthermore, the constant development of brighter fluorescent proteins with narrower excitation and emission spectra is expected to further push the boundaries of time resolution of live imaging as well as optimize multicolor imaging. Finally, the simultaneous visualization of mRNAs, their translation state and the fate of the encoded proteins in living cells remains currently still out of reach and will require additional technological improvement. Recent methods have been developed for mammalian cells and for drosophila, but to this date, the microbiology field still has only limited tools (reviewed in [2]).

Across kingdoms of life, a common theme is emerging: molecules, organelles and subcellular compartments are organized and interconnected. The function of such organization still awaits to be elucidated, and the development of sensitive high-resolution methods is an essential prerequisite. Approaches to quantitatively assess the gene expression cycle in individual cells have the potential to reveal whether different gene expression steps are spatiotemporally coordinated and how does this contribute to cellular adaptation and physiology. Significant efforts still need to be done in the microbiology filed, whereby less imaging technology development has been achieved over the past decades. This could have far-reaching consequences on the field of molecular biology, biotechnology and medical microbiology.
